# Patient survival and risk of death after prostate cancer treatment in the Brazilian Unified Health System

**DOI:** 10.1590/S1518-8787.2017051006766

**Published:** 2017-05-08

**Authors:** Sonia Faria Mendes Braga, Mirian Carvalho de Souza, Raphael Romie de Oliveira, Eli Iola Gurgel Andrade, Francisco de Assis Acurcio, Mariangela Leal Cherchiglia

**Affiliations:** I Programa de Pós-Graduação em Saúde Pública. Faculdade de Medicina. Universidade Federal de Minas Gerais. Belo Horizonte, MG, Brasil; II Instituto Nacional do Câncer José Alencar Gomes da Silva. Divisão de Epidemiologia Clínica. Rio de Janeiro, RJ, Brasil; III Faculdade de Medicina. Universidade Federal de Minas Gerais. Belo Horizonte, MG, Brasil; IV Departamento de Medicina Preventiva e Social. Faculdade de Medicina. Universidade Federal de Minas Gerais. Belo Horizonte, MG, Brasil; V Departamento de Farmácia Social. Faculdade de Farmácia. Universidade Federal de Minas Gerais. Belo Horizonte, MG, Brasil

**Keywords:** Prostatic Neoplasms, Mortality, Risk Factors, Survivorship (Public Health), Men’s Health, Unified Health System, Neoplasias da Próstata, Mortalidade, Fatores de Risco, Sobrevida, Saúde do Homem, Sistema Único de Saúde

## Abstract

**OBJECTIVE:**

Analyze the probability of specific survival and factors associated with the risk of death of patients with prostate cancer who received outpatient cancer treatment in the Brazilian Unified Health System, Brazil.

**METHODS:**

Retrospective cohort study using the National Database of Oncology, developed through the deterministic-probabilistic pairing of health information systems: outpatient (SIA), hospital (SIH) and mortality (SIM). The probability of overall and specific survival was estimated by the time elapsed between the date of the first ambulatory treatment, from 2002 to 2003, until the patient’s death or the end of the study. Fine and Gray’s model of competing-risks regression was adjusted according to the variables: age of diagnostic, region of residence, tumor clinical staging, type of outpatient cancer treatment and hospitalization in the assessment of factors associated with risk of patient death.

**RESULTS:**

Of 16,280 patients studied, the average age was 70 years, approximately 25% died due to prostate cancer and 20% for other causes. The probability of overall survival was 0.50 (95%CI 0.49–0.52) and the specific was 0.70 (95%CI 0.69–0.71). The factors associated with the risk of patient death were: stage III (HR = 1.66; 95%CI 1.39–1.99) and stage IV (HR = 3.49; 95%CI 2.91–4.18), chemotherapy (HR = 2.34; 95%CI 1.76–3.11) and hospitalization (HR = 1.6; 95%CI 1.55–1.79).

**CONCLUSIONS:**

The late diagnosis of the tumor, palliative treatments, and worse medical condition were factors related to the worst survival and increased risk of death from prostate cancer patients in Brazil.

## RESUMO

**OBJETIVO:**

Analisar a probabilidade de sobrevida específica e os fatores associados ao risco de óbito dos pacientes com câncer de próstata, que receberam tratamento oncológico ambulatorial no SUS, Brasil.

**MÉTODOS:**

Estudo de coorte retrospectivo utilizando a Base Nacional em Oncologia, desenvolvida por meio de pareamento determinístico-probabilístico dos sistemas de informação de saúde: ambulatorial (SIA), hospitalar (SIH) e de mortalidade (SIM). A probabilidade de sobrevida global e específica foi estimada pelo tempo decorrido entre a data do primeiro tratamento ambulatorial, entre 2002 e 2003, até o óbito dos pacientes ou fim do estudo. O modelo de regressão de riscos competitivos de Fine e Gray foi ajustado segundo as variáveis: idade ao diagnóstico, região de residência, estadiamento clínico do tumor, tipo de tratamento oncológico ambulatorial e internação na avaliação dos fatores associados ao risco de óbito dos pacientes.

**RESULTADOS:**

Dos 16.280 pacientes estudados, a idade média foi de 70 anos, cerca de 25% foi a óbito devido ao câncer de próstata e 20% por outras causas. A probabilidade de sobrevida global foi de 0,50 (IC95% 0,49–0,52) e a específica 0,70 (IC95% 0,69–0,71) . Os fatores associados ao risco de óbito dos pacientes foram: estádio III (HR = 1,66; IC95% 1,39–1,99) e estágio IV (HR = 3,49; IC95% 2,91–4,18), tratamento quimioterápico (HR = 2,34; IC95% 1,76–3,11) e internação (HR = 1,6; IC95% 1,55–1,79).

**CONCLUSÕES:**

O diagnóstico tardio do tumor, tratamentos não curativos e pior condição clínica foram fatores relacionados à pior sobrevida e ao maior risco de óbito dos pacientes com câncer próstata no Brasil.

## INTRODUCTION

According to estimates, in 2012 prostate cancer caused more than 300,000 deaths. It was the second type of cancer with greater incidence among men, with more than one million new cases in the world^a^.

In Brazil, an estimated 70,000 new cases of prostate cancer were diagnosed in 2015. This is the most incident neoplasm in all regions, excluding non-melanoma skin tumors, with highest rates in the South and Southeast regions. Following the global trend, the increase in incidence rates in the country was due to the increase in life expectancy, improved diagnostic methods and notification systems, and the spread of prostate-specific antigen (PSA), and digital rectal examination in the diagnosis of this neoplasm^b^. The age-adjusted mortality rate shows an upward curve similar to the incidence, but to a lesser magnitude, from 7.44/100,000 men in 1980 to 14.06/100,000 men in 2013^c^.

Some of the important risk factors for the development of prostate cancer are: age, because this cancer usually affects men over the age of 50 years and the risk of illness increases with advancing age; family history of prostate cancer, as individuals with close relatives who have cancer have almost twice the risk of developing this neoplasm compared to the general population; and skin color, because larger incidence rates are observed and tumors are more aggressive tumors in black individuals^4^ ^,^ ^22^.

In general terms, advances in diagnosis and treatments resulted in a higher proportion of patients being cured or surviving longer with cancer, making survival a relevant public health issue. In addition, survival can be an indicator to assess results in the use of oncology health services, since survival rates can help estimate the system’s capacity for providing high-quality patient care^2^.

Since prostate cancer affects older people more frequently, in general, these individuals already have comorbidities at diagnosis. Thanks to that, the risk of death by this neoplasm can be difficult to observe due to the presence of another event (death by other causes)^9^ ^,^ ^13^ ^,^ ^18^. The event that hinders or modifies the possibility to observe the event of interest is a competitive risk. The use of specific techniques to analyze competitive risks ensures that the results are not biased and can be interpreted correctly^19^.

This study’s objective is to analyze the specific probability of survival and factors associated with the risk of death of patients with prostate cancer who received outpatient cancer treatment in the Brazilian Unified Health System (SUS).

## METHODS

This is a retrospective cohort study whose data source was the National Database in Oncology (Base Onco), created in the “Economical-Epidemiological Evaluation of Cancer Treatment in the Brazilian Unified Health System (SUS) between 2000 and 2006 in Brazil” project, and carried out by the Group of Health Economics at Universidade Federal de Minas Gerais. This database was developed through the technique of deterministic-probabilistic relationship between data information systems: High-Complexity Procedures Permit/Cost (APAC-Oncologia), Mortality Information System (SIM) and SUS Hospital Information System (SIH), in order to enable the cohort follow-up^1^. The methodological procedure was the same used for the construction and quality evaluation of the data relationship in the National Database for Renal Therapy Substitution (Base TRS), as described in Cherchiglia et al.^3^

Of the prostate cancer patients identified in the Base Onco, this analysis includes those with: (i) date of the first outpatient cancer treatment recorded in the first APAC from January 1, 2002 to December 31,2003, because the pairing of the bases used SIM’s basis from 2002 to 2008 in order to complete patient follow-up; (ii) age between 20 and 100 years, since the treatment protocol for younger individuals is usually different, even in regards to authorization procedures by SUS^d^; and (iii) clinical stage I to IV, because tumors in stage 0 (*in situ*) are often difficult to differentiate histologically and classified as benign or uncertain whether benign or malign^2^.

The time elapsed from the date of the first oncological outpatient procedure to the date of death by prostate cancer or other causes or the final date of follow-up were studied (December 31, 2008). The associated factors assessed were: age group at the start of the follow-up, the region of residence at first record, the tumor’s clinical staging at diagnosis, type of treatment (radiation or chemotherapy) and hospitalization in SUS.

The patient’s profile included in the study was described with the aid of position and dispersion proportions and measures. For the overall survival analysis, we considered as an event of interest the death regardless of cause and censored patients not found in the SIM database until December 31, 2008 (non-informative censorship). For the analysis of specific survival, we considered as event of interest the death with ICD-10 of prostate cancer (C61) and which had, in one of the lines describing the cause of death on the death certificate: root cause, line A, line B or line C. As a competitive event, we considered death by causes not related to prostate cancer, also described in the lines: root cause, line A, line B and line C. We censored patients not found in the SIM database until December 31, 2008 (who had not experienced the event of interest or competitive event – informational censorship).

To estimate the probability of overall survival for the minimum period of five years we applied the Kaplan-Meier method. Specific survival functions were estimated using Fine and Gray’s competitive risks model^6^, which lists the covariates of accumulated incidence function taking into account the main event (death by prostate cancer) and the competitive event (death by other causes). Gray’s test^8^ was used to check the equality of accumulated incidences between the categories of the factors evaluated in the presence of competitive risks. The factors with p-value associated with the risk measure lower than 0.10 were included in the multiples model. Fine and Gray’s model of competing-risks regression was used in the assessment of factors associated with specific survival of patients diagnosed with prostate cancer. The statistical procedures were implemented in the free software R, version 3.1.3, using the libraries: survival, foreign, chron, cmprsk, and risk Regression^e^.

The Research Ethics Committee of the Universidade Federal de Minas Gerais (Protocol ETIC 072/09, April 29, 2009) approved this project.

## RESULTS

Of the 651,328 patients in Base Onco, covering the period from 2000 to 2006, 19,700 were diagnosed with prostate cancer and entered outpatient cancer treatment between 2002 and 2003 in SUS. Of those we excluded the following for data inconsistency: 532 patients did not have date of birth; nine patients registered as female; one patient with cancer procedure incompatible with prostate cancer treatment; 1,871 patients with treatment date previous to 2002; 290 patients with treatment date prior to the date of diagnosis and 66 patients with date of death prior to the date of diagnosis. After this process, 16,931 patients remained on the database.

According to the inclusion criteria established for this study, we excluded six patients under 20 years of age, three over 100 years of age and 642 with tumors at stage 0. At the end of the selection procedures, we analyzed the data of 16,280 individuals in outpatient cancer treatment for prostate cancer in SUS between 2002 and 2003, in Brazil. Of this total, we observed 8,914 censures, 4,037 prostate cancer deaths (the event of interest) and 3,329 deaths from other causes (competitive event).

The average age of patients was 70.5 years with a standard deviation (SD) of 8.7 years and median of 71 years. We observed that more than 75% of the patients were between 60 and 79 years old at the beginning of the outpatient treatment, and in the region of residence category the small proportion of residents in the Northern region is of notice. About 60% of patients were diagnosed in more advanced stages of the disease (stages III and IV), had chemotherapy (62%) and over 80% were not hospitalized in SUS (Table 1).

**Table 1:** Demographic, clinical, and treatment characteristics of patients diagnosed with prostate cancer between 2002–2003 in SUS, Brazil.

Characteristics studied	n	%
Total	16,280	100
Age group at the start of the follow-up (years)		
20–59	1,739	10.7
60–69	5,121	31.5
70–79	7,148	43.9
≥ 80	2,272	14.0
Region of residence		
Southeast	8,711	53.5
South	2,677	16.4
Midwest	763	4.7
North	439	2.7
Northeast	3,690	22.7
Clinical stages		
Stage I	1,194	7.3
Stage II	5,705	35.1
Stage III	4,149	25.5
Stage IV	5,232	32.1
First outpatient treatment		
Radiotherapy	6,175	37.9
Hormone therapy – First line	7,298	44.8
Hormone therapy – Second line	2,666	16.4
Castration-resistant chemotherapy	141	0.9
Hospitalized in SUS		
No	13,124	80.6
es	3,156	19.4
Number of hospitalizations		
0	13,124	80.6
1	1,919	11.8
2	687	4.2
3	256	1.6
≥ 4	294	1.8
Death during the study period		
No	8,914	54.8
Yes, by prostate cancer	4,037	24.8
Yes, by other causes	3,329	20.5

Source: Base Onco, 2006.

The time between the diagnosis of prostate cancer and the start of outpatient cancer treatment had an average of five months and median of three months, with high SD (6.0 months). The patients were followed for up to 83 months, with the average follow-up of 51 months (SD = 25.6 months) (data not presented in table).

Patients with prostate cancer had an estimated probability of survival up to 83 months of 0.50 (95%CI 0.49–0.52) for overall survival and 0.70 (95%CI 0.69–0.71) for specific survival. The odds of survival decreases as the patient’s age advances, but specific survival under 60 years of age was lower than those of older adults were. The Southern region presented the lowest probabilities of specific and overall survival. The overall survival in stage IV is 45% lower than in stage I. Patients treated with chemotherapy had the worst overall and specific survival compared to those treated with radiotherapy. Hospitalized patients showed worse prognosis (Table 2).

**Table 2:** The probability of survival (PS) at up to 83 months in patients diagnosed with prostate cancer and treated between 2002–2003 in SUS, Brazil.

Characteristics studied	Probability of survival			

	Global		Specific	
	
	PS	95CI%	PS	95CI%
Total	0.50	0.49–0.52	0.70	0.69–0.71
Age group at the start of the follow-up (years)				
20–59	0.56	0.50–0.62	0.65	0.58–0.72
60–69	0.56	0.54–0.58	0.71	0.69–0.73
70–79	0.50	0.49–0.52	0.72	0.71–0.74
≥ 80	0.34	0.31–0.38	0.63	0.60–0.66
Region of residence				
Southeast	0.51	0.50–0.53	0.72	0.71–0.73
South	0.45	0.42–0.49	0.62	0.58–0.67
Midwest	0.53	0.49–0.57	0.68	0.64–0.71
North	0.53	0.47–0.59	0.71	0.65–0.77
Northeast	0.51	0.48–0.54	0.71	0.69–0.73
Clinical stages				
Stage I	0.65	0.61–0.68	0.85	0.82–0.88
Stage II	0.62	0.60–0.63	0.82	0.80–0.84
Stage III	0.51	0.48–0.54	0.72	0.70–0.75
Stage IV	0.35	0.33–0.37	0.51	0.49–0.53
First outpatient treatment				
Radiotherapy	0.62	0.60–0.64	0.80	0.78–0.82
Hormone therapy – First line	0.45	0.43–0.46	0.64	0.63–0.66
Hormone therapy – Second line	0.42	0.39–0.46	0.64	0.61–0.67
Castration-resistant chemotherapy	0.24	0.17–0.32	0.46	0.38–0.56
Hospitalized in SUS				
No	0.53	0.52–0.55	0.73	0.72–0.74
Yes	0.38	0.36–0.40	0.57	0.55–0.59
Number of hospitalizations				
0	0.53	0.52–0.55	0.73	0.72–0.74
1	0.44	0.41–0.47	0.63	0.60–0.66
2	0.35	0.29–0.41	0.54	0.50–0.58
3	0.29	0.23–0.35	0.46	0.40–0.54
≥ 4	0.14	0.10–0.19	0.30	0.24–0.37

Source: Base Onco, 2006.

The cumulative incidence functions showed that by using Fine and Gray’s competitive risks model, the probability of death by other causes was lower than the probability of death by prostate cancer during the whole follow-up. However, the probability of global death was close to the probability of death by prostate cancer (Figure) when using Kaplan-Meier without accounting for competitive events.

**Figure:**
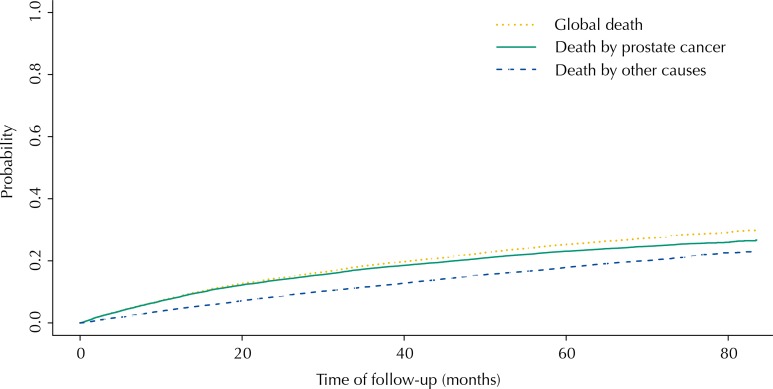
Cumulative incidence function for patients diagnosed with prostate cancer between 2002 and 2003 in SUS.

Rate analysis for prostate cancer death risk indicated that, when adjusting for other factors in the final model, the patients in the age group of 60 to 80 years or more have a lower risk of death when compared to younger patients; patients in stage IV have a risk of death 3.49 times higher compared to patients in stage I; hormone therapy has similar risk rates regardless of treatment line. Patients who had chemotherapy-resistant to hormonal castration had a risk of death 2.34 times greater than those who did radiotherapy. Hospitalization increased the risk of death by prostate cancer (Table 3).

**Table 3:** Gross estimates and adjusted risk of death for patients diagnosed with prostate cancer and treated between 2002–2003 in SUS, Brazil.

Characteristics studied	Gross HR	Adjusted HR
	
	(95%CI)^a^	(95%CI)^a,b^
Age group at the start of the follow-up (years)^c,d^		
20–59	1.00	1.00
60–69	0.81 (0.73–0.90)	0.85 (0.77–0.94)
70–79	0.77 (0.69–0.85)	0.82 (0.74–0.91)
≥ 80	0.98 (0.88–1.11)	0.96 (0.85–1.08)
Region of residence^c^		
Southeast	1.00	1.00
South	1.42 (1.30–1.54)	1.12 (1.03–1.22)
Midwest	1.26 (1.09–1.45)	0.93 (0.80–1.07)
North	1.00 (0.82–1.23)	1.02 (0.83–1.25)
Northeast	0.99 (0.92–1.08)	0.99 (0.91–1.08)
Clinical stages^c,d^		
Stage I	1.00	1.00
Stage II	1.20 (1.00–1.43)	1.15 (0.96–1.37)
Stage III	1.90 (1.59–2.26)	1.66 (1.39–1.99)
Stage IV	4.42 (3.73–5.23)	3.49 (2.91–4.18)
First outpatient treatment^c,d^		
Radiotherapy	1.00	1.00
Hormone therapy – First line	2.04 (1.89–2.20)	1.28 (1.17–1.40)
Hormone therapy – Second line	1.95 (1.78–2.15)	1.39 (1.25–1.55)
Castration-resistant chemotherapy	4.21 (3.21–5.52)	2.34 (1.76–3.11)
Hospitalized in SUS^c,d^		
No	1.00	1.00
Yes	1.88 (1.76–2.01)	1.67 (1.55–1.79)

Source: Base Onco, 2006.

^a^ HR = hazard ratio*.*

^b^ Values adjusted by age group at the start of the follow-up, region of residence, clinical staging, first outpatient treatment and hospitalization in SUS.

^c^ P-value in Grey’s test lower than 0.10 in the gross analysis.

^d^ P-value in Grey’s test lower than 0.05 in the adjusted analysis.

## DISCUSSION

This study used Base Onco, constituted through the data grouping of the main health information systems in Brazil, enabling us to understand the profile and specific survival of prostate cancer patients diagnosed and treated in SUS, between 2002 and 2003.

The results showed that prostate cancer affects mainly men between the ages of 70 and 79 years, diagnosed in late clinical stage (stages III and IV) who, after the diagnosis, waited about five months to start cancer treatment, which was usually chemotherapy. In addition, 25% of patients died due to cancer and 20% due to other causes. These results have support in the literature because several studies show that prostate cancer affects older individuals, who coexist with other diseases beyond the tumor, which affects these patients survival^9^ ^,^ ^13^ ^,^ ^18^.

In regards to the probability of survival, the results showed that the probability of overall and specific survival of patients in nearly seven years was 0.50 and 0.70, respectively. Migowski and Silva^16^ studied a hospital cohort, the National Cancer Institute (INCA) in Rio de Janeiro, composed of 258 patients between 1990 and 1999, and found a specific survival probability of 0.88 in five years and 0.71 in 10 years. Another study, developed by Pirajá et al.^20^ (Teresina, State of Piauí) and that assessed a cohort of 71 patients, found a specific survival probability of 0.78 at five years. In Migowski and Silva’s^16^ study, eligible patients were at stages I and II, and therefore showed better odds of survival than the patients in this investigation. In the Pirajá et al.^20^ study, eligible patients were in stages I to IV, similar to patients in this study, however, they showed better survival.

The lowest specific survival probability found by this research in regards to hospital-based studies may be due to the profile of the population served, as well as the contexts of the country’s hospitals and their ability to produce health results that are quite specific to the studied population and that do not allow generalization for the population at large^7^. The studies using records of health information systems refer to all cases of cancer identified in a population and geographically distributed, and may even reflect the quality of health services for patient treatment^10^ ^,^ ^12^ ^,^ ^13^ ^,^ ^18^.

Nguyen-Nielsen et al.^18^ investigated the overall survival at 5 years in prostate cancer patients in Denmark from 2000 to 2011, which ranged from 0.43 to 0.65. The authors claimed that the survival probability improved significantly in the periods studied, but that prostate cancer is often complicated by comorbidities or other preexisting conditions, such as cardiovascular diseases, cerebrovascular disease, diabetes and other primary cancers related to age, which end up competing for causes of death with the cancer itself.

In this research, when we estimated the odds of survival at up to 83 months according to the categories of analysis studied, the results showed that there was a reduction in overall and specific survival as the clinical staging advances, when there were chemotherapy treatment and hospitalization once or more times. This reinforces that the health system needs to offer, in all levels of care, individuals a timely diagnosis and treatment of the disease, mainly for patients with urinary symptoms, avoiding more aggressive treatments and a prognostic worsening of the disease^f^. It is noticeable that, in regards to age, the particular survival of patients was lower in younger age groups than in the older ones (with the exception of the very elderly). Some studies have examined the worse prognosis of the disease in younger individuals as a possible result of the diagnosis in more severe stages^15^.

In survival analysis, the probability of patients experiencing the event of interest at any given time is the primary focus. When the data consist of patients who experience the event of interest whereas others are censored (non-informative censorship), the events are independent. In other times, the patient may experience another event, other than the event of interest, that is considered risk competitive events (dependent, and censures are considered informative)^11^ ^,^ ^19^ ^,^ ^21^. In the present study, when investigating the other causes of death, we found that the main groups of diseases described were infectious diseases (pulmonary and urinary), metabolic diseases (diabetes mellitus), circulatory system diseases (hypertension and stroke) and respiratory diseases (asthma and chronic obstructive pulmonary disease), which are common in advanced age. This scenario probably influenced the estimates as to the probability of specific survival showed, given that about 90% of the patients studied were over 60 years old.

Among the factors associated with risk of death in patients diagnosed with prostate cancer in Brazil, older age groups showed lower death risk than younger groups. Migowski and Silva^16^ considered the existence in the past of diagnostic bias among youngsters, when only symptomatic cases were diagnosed, which increased the detection risk of more aggressive tumors. However, Lin et al.’s^12^ study, using records from the Surveillance, Epidemiology and End Results (SEER) performed in the USA between 1988 and 2003, showed that the overall survival of patients decreases with advancing age. Yet, the authors showed that younger men had a higher risk of death for a specific cause (prostate cancer), and especially a worse prognosis for the disease when compared to older patients.

In this study, patients in stage IV showed a much higher risk of death in relation to earlier stages. The stage at diagnosis is a classic prognostic factor in oncology^14^. A longitudinal study^10^ performed in two cities in China assessed the trend of mortality, incidence and survival of prostate cancer patients between 2000 and 2009 and showed that one of the factors that influenced the probability of survival the most was the stage at diagnosis – patients in stages III and IV had their death risk tripled in relation to those in stage I. However, a study by Muralidhar et al.^17^ evaluating 66,817 patients with stage IV prostate cancer between 1973 and 2011, using data from SEER, showed that specific survival at 5, 10 and 15 years has improved over time. According to authors^17^, it is up to doctors to provide these patients, even at that stage, appropriate follow-up, hoping for a better prognosis over time.

As for treatment, there was a higher risk of death for patients whose therapeutic schemes involved chemotherapy. Radiotherapy and surgery are the treatments for cancer in the early stages of the disease, hormone therapy is for patients who do not present locoregional advanced metastasis, and castration-resistant chemotherapy is for advanced disease with metastases in the distance^g^. DiBlasio et al.^5^ analyzed the overall and specific survival in men treated with hormone therapy of first and second line for 20 years in the United States and showed that treatments prolonged the patients’ survival, but the competitive risks caused by other diseases or comorbidities contributed to reducing the survival in both treatment groups. Hoffman et al.^9^ investigated the specific survival in older patients with stages III and IV cancer comparing those who received only brachytherapy and those who received the combined treatment (hormone therapy, external beam radiotherapy, brachytherapy). The authors found a lower risk of death for those who received the combined treatment and showed that patients, even if elderly, may benefit from treatments that are more aggressive. However, it depends on whether they are healthy or if there are other comorbidities that coexist with the tumor.

Hospitalized patients presented a higher risk of death by cancer, probably because they presented a greater number of complications, comorbidities or worse clinical conditions, especially for being older adults. A study developed in France, in 2014, by Tuppin et al.^23^ using the National Health Insurance Information System (SNIIRAM), investigated the health results, after the diagnosis of prostate cancer in terms of mortality rates, treatments and their adverse effects in men between 50 to 69 years of age. The authors reported high survival rates at two years, but a high frequency of adverse effects related to the treatments. The complications found were due to urinary and erectile dysfunction disorders, depending on the type of treatment, and are defined based on at least one hospitalization and the presence of surgical procedures to treat such complications.

Limitations on the use of a database of administrative origin must be mentioned, as the identification of clinical information gaps, difficulty of coding procedures, database billing character, lack of socioeconomic and demographic variables that characterize the individual and also on the use of a death certificate as a source for the description of cause of death. In addition, working with administrative databases it is not possible to include patients who had isolated surgery as a treatment because the pairing with SIH was conducted through patients in outpatient cancer treatment in SUS.

The assessment of specific survival and the factors associated with the risk of death in this investigation allowed us to show that individuals are diagnosed late and that there is a long period before starting treatment after diagnosis. Consequently, they receive more palliative treatment to healing treatment. This may reflect difficulties in the use of health services for cancer preventive examinations (PSA and digital rectal examination), as well as to access cancer assistance and timely treatment. In addition, because this cancer tends to affect older adults in higher proportions, they should be monitored not only for cancer, but also for other diseases that can compromise a more favorable prognosis of the disease, and, consequently, the probability of survival for these individuals.

## Footnotes

a Ferlay J, Soerjomataram I, Ervik M, Dikshit R, Eser S, Mathers C, Rebelo et al. GLOBOCAN 2012: cancer incidence and mortality worldwide: v.10. Lyon: International Agency for Research on Cancer; 2013 [cited 2015 Sept 24]. (IARC CancerBase, No. 11). Available from: http://globocan.iarc.fr

b Instituto Nacional do Câncer José Alencar Gomes da Silva. Estimativa 2014: incidência de câncer no Brasil - 2013. Rio de Janeiro: INCA; 2014 [cited 2014 Oct 19]. Available from: http://www.inca.gov.br/estimativa/2014/

c Instituto Nacional do Câncer José Alencar Gomes da Silva (INCA). Atlas de mortalidade por câncer Rio de Janeiro: INCA; 2013 [cited 2015 Aug 15]. Available from: https://mortalidade.inca.gov.br/MortalidadeWeb/

d Ministério da Saúde (BR), Secretaria de Atenção à Saúde, Instituto Nacional do Câncer José Alencar Gomes da Silva. Sistema de Informações Ambulatoriais do SUS (SIA/SUS): manual de bases técnicas em Oncologia. Brasília (DF); 2006 [cited 2013 May 29]. Available from: http://bvsms.saude.gov.br/bvs/publicacoes/inca/manual_oncologia_14edicao.pdf

e R Core Team. The R Project for Statistical Computing: a language and environment for statistical computing. Vienna: R Foundation for Statistical Computing; 2015 [cited 2016 Feb 22]. Available from: https://www.R-project.org

f Instituto Nacional do Câncer José Alencar Gomes da Silva (INCA). Monitoramento das ações e controle do câncer da próstata. Inf Detec Precoce Bol. 2014;5(2) [cited 2015 Sept 30]. Available from: http://www.saude.pi.gov.br/ckeditor_assets/attachments/531/boletim_informativo_inca_-_cancer_de_prostata__2_.pdf

g Dall’Oglio MF, Crippa A, Faria EF, Cavalhal GF, et al. Diretrizes de câncer de próstata. Rio de Janeiro: Sociedade Brasileira de Urologia; 2011 [cited 2015 Jan 15]. Available from: http://sbues.org.br/diretrizes/cancer_prost ata.pdf
